# The emergence of piRNAs against transposon invasion to preserve mammalian genome integrity

**DOI:** 10.1038/s41467-017-01049-7

**Published:** 2017-11-10

**Authors:** Christina Ernst, Duncan T. Odom, Claudia Kutter

**Affiliations:** 1 University of Cambridge, Cancer Research UK Cambridge Institute, Robinson Way, Cambridge, CB2 0RE UK; 2 Department of Microbiology, Tumor and Cell Biology, Science for Life Laboratory, Karolinska Institute, Nobels väg 16, 171 77 Stockholm, Sweden

## Abstract

Transposable elements (TEs) contribute to the large amount of repetitive sequences in mammalian genomes and have been linked to species-specific genome innovations by rewiring regulatory circuitries. However, organisms need to restrict TE activity to ensure genome integrity, especially in germline cells to protect the transmission of genetic information to the next generation. This review features our current understandings of mammalian PIWI-interacting RNAs (piRNAs) and their role in TE regulation in spermatogenesis. Here we discuss functional implication and explore additional molecular mechanisms that inhibit transposon activity and altogether illustrate the paradoxical arms race between genome evolution and stability.

## Abstract (web summary)

Transposable elements can be activated during germ cell maturation, potentially leading to genome instability and rewiring of the genetic circuitry. In this review, the authors discuss how the piRNA machinery suppresses these elements to ensure accurate spermatogenesis.

## Introduction

Repetitive sequences make up a large proportion of mammalian genomes, with up to two thirds of the human genome being repeat-derived^1^. A subfraction of these repeats are mobile elements, also termed transposable elements (TEs), that play crucial roles in driving genome evolution by fueling the development of new genes, introducing novel immune strategies such as V(D)J recombination, and rewiring gene regulatory circuitries^2^. However, such plasticity comes at the price of causing potential deleterious effects as a result of uncontrolled retrotransposition and therefore requires a tight handling of TEs by their host cells^3^. This is especially important in cells contributing to the germline, to ensure the integrity of the genome that is passed on to the next generation. To this effect, an additional layer of retrotransposon control has evolved in the metazoan germline that is based on small RNA-mediated recognition of TE transcripts called the piRNA pathway.

Active retrotransposition is more frequent in germ cells since the epigenetic reprogramming that primes these cells for totipotency also results in the derepression of TEs^4^. A requirement for the successful retrotransposition of a TE is active cell divisions^5^. Hence, the burden is heavier during male germ cell development in mammals, which is marked by continuous waves of spermatogenesis throughout the life span, compared to female germ cells of which a defined number arrests in meiosis I during embryonic development and only matures after the onset of sexual maturity^6^.

In this review, we will discuss the role of PIWI-interacting RNAs (piRNAs) throughout mammalian, mostly mouse, spermatogenesis and their interplay with transposable elements and briefly touch on additional silencing mechanisms controlling the activity of transposable elements.

## Regulatory dynamics of mouse spermatogenesis

**Fig. 1:**
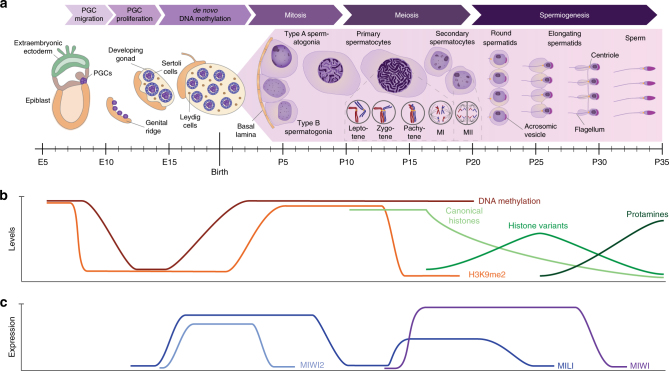
Male germ cell nomenclature and developmental dynamics of mouse spermatogenesis. **a** Gametogenesis starts during embryonic development when primordial germ cells (PGCs) are defined and migrate to the genital ridge to form the gonads. Spermatogenesis initiates shortly after birth in synchronized waves. At 10 days post birth (P10), spermatogonial stem cells differentiate into primary spermatocytes that are committed to undergo meiosis. Two consecutive cell divisions (meiosis I and II (MI and MII)) without an intermediate S-phase result in the production of haploid gametes that are called round spermatids. These cells can be found as early as P20 and then undergo spermiogenesis during which the cells elongate and develop sperm-specific structures such as the acrosome and the flagellum to form mature sperm cells. **b** The process of gametogenesis is associated with extensive epigenetic reprogramming accompanied by drastic changes in DNA methylation and histone modifications such as H3K9me2. During later stages of spermatogenesis, global changes in histone composition and finally a histone-to-protamine exchange result in chromatin compaction. **c** The three PIWI proteins encoded in the mouse genome show very specific expression profiles throughout spermatogenesis and reflect functionally distinct aspects of the piRNA pathway at different stages of spermatogenesis

Gametogenesis is a complex process that starts as early as embryonic day 7.5 (E7.5) with the emergence of primordial germ cells (PGCs) that migrate to and populate the genital ridges at E10.5–E11.5^7^ (Fig. 1a). Migratory PGCs experience various epigenetic changes, such as global erasure of histone H3K9me1/2, which is linked to decreased expression of the H3K9 methyltransferase G9a-like protein, as well as an increase in H3K27me3 and various histone variants (Fig. 1b)^7^. PGCs arrive at the genital ridge during midgestation at E10.5–11.5, where they continue their reprogramming resulting in a global loss of DNA methylation^8^. Once PGCs are residing within the gonads, sexual dimorphism occurs around E11.5 and male PGCs continue to proliferate in the gonads until they enter mitotic arrest at E14 (at which point ~25.000 PGCs are found in each gonad)^9^. Male PGCs remain mitotically arrested until postnatal day 2 (P2), during which time *de novo* DNA methylation and the establishment of paternal imprints takes place^10^. This process is mediated by the *de novo* DNA methyltransferases DNMT3A and DNMT3B as well as their catalytically inactive interaction partner DNMT3L^11^. DNMT3L is essential for spermatogenesis by guiding *de novo* DNA methylation and binding to unmethylated histone H3 lysine 4 tails^12^.

Male PGCs resume cell division shortly after birth and give rise to type-A spermatogonia, which are found on the basement membrane of seminiferous tubules. These cells have self-renewing potential but also produce type-B spermatogonia, which can enter meiosis and become spermatocytes^13^. After several rounds of mitotic divisions from P2 onwards, the first meiosis is initiated in a synchronous manner around P10. As cells progress through spermatogenesis, they move away from the basement membrane toward the lumen of seminiferous tubules and enter pre-leptotene/leptotene, zygotene, pachytene, and diplotene stages on P10, 12, 14, and 17, respectively^13^. Prophase of meiosis I is unique in that it promotes transfer of genetic information between the parental genomes via crossovers. To allow meiotic crossovers, homologous chromosomes synapse during the zygotene stage and form bivalents^14^. This is mediated by DNA double-strand breaks (DSBs) that are specifically induced by SPO11^15^ and enable homologous recombination (HR) between non-sister chromatids of homologous chromosomes. Crossovers are essential for proper chromosome synapsis and occur in hotspots across mammalian genomes, directed by PRDM9^16–18^. Failures in *de novo* DNA methylation have been shown to cause mislocalization of crossovers resulting in spermatogenic arrest at the pachytene stage^19^. When reaching the diplotene stage of prophase I, homologous chromosomes begin to separate but are still attached to each other, thereby forming the characteristic structures called chiasmata. Eventually, bivalents are arranged on the metaphase plate and homologous chromosomes are separated to opposite poles followed by cytokinesis ending the first meiotic division.

The second meiotic division follows promptly and generates the first set of round spermatids to be found at P20. Round spermatids then undergo a complex differentiation program called spermiogenesis, during which they form sperm-specific structures such as the acrosome and flagellum and undergo nuclear compaction^20^. The latter is achieved via genome-wide replacement of histones with transition proteins at first, followed by protamines, which results in up to a 20 times more condensed structure due to the basic character of these proteins^21^. To allow the transition, a loose chromatin structure is adopted and mediated in part by the incorporation of various testis-specific histone variants, which leads to a global transcriptional activation including TEs^22–24^. Histone replacement, however, was shown to be incomplete with some genomic regions retaining their histones including epigenetic modifications to potentially prime these regions for early transcription after fertilization^25–27^.

The different waves of epigenetic reprogramming and global remodeling of the genome allow repetitive elements to become transcriptionally active in the male germline at several developmental time points. To prevent these repetitive elements from causing genome instability, additional control mechanisms are required during spermatogenesis.

## Mouse PIWI proteins

The mouse male germline expresses three proteins of the P-element-induced wimpy testis (PIWI) family: MILI, MIWI, and MIWI2, all of which are essential for spermatogenesis and male fertility^28–30^. Each PIWI protein exhibits a unique expression profile throughout spermatogenesis (Fig. 1c) and associates with a defined population of small non-coding RNAs called PIWI-interacting RNAs (piRNAs)^31–34^. PIWI proteins are members of the highly conserved Argonaute family and are characterized by their PIWI, MID and PAZ domains^35^. The PIWI domain is essential for the endonucleolytic slicer activity of PIWI proteins^36^, while the MID and PAZ domains function in anchoring the 5′ and 3′ ends of piRNAs, respectively^37–39^. In mice, PIWI proteins and piRNAs are almost exclusively found in the male germline, with low expression levels of MILI and piRNAs in oocytes^30, 40, 41^ and some recent reports indicating their expression in somatic cells^42, 43^.

## Primary piRNA biogenesis

**Fig. 2:**
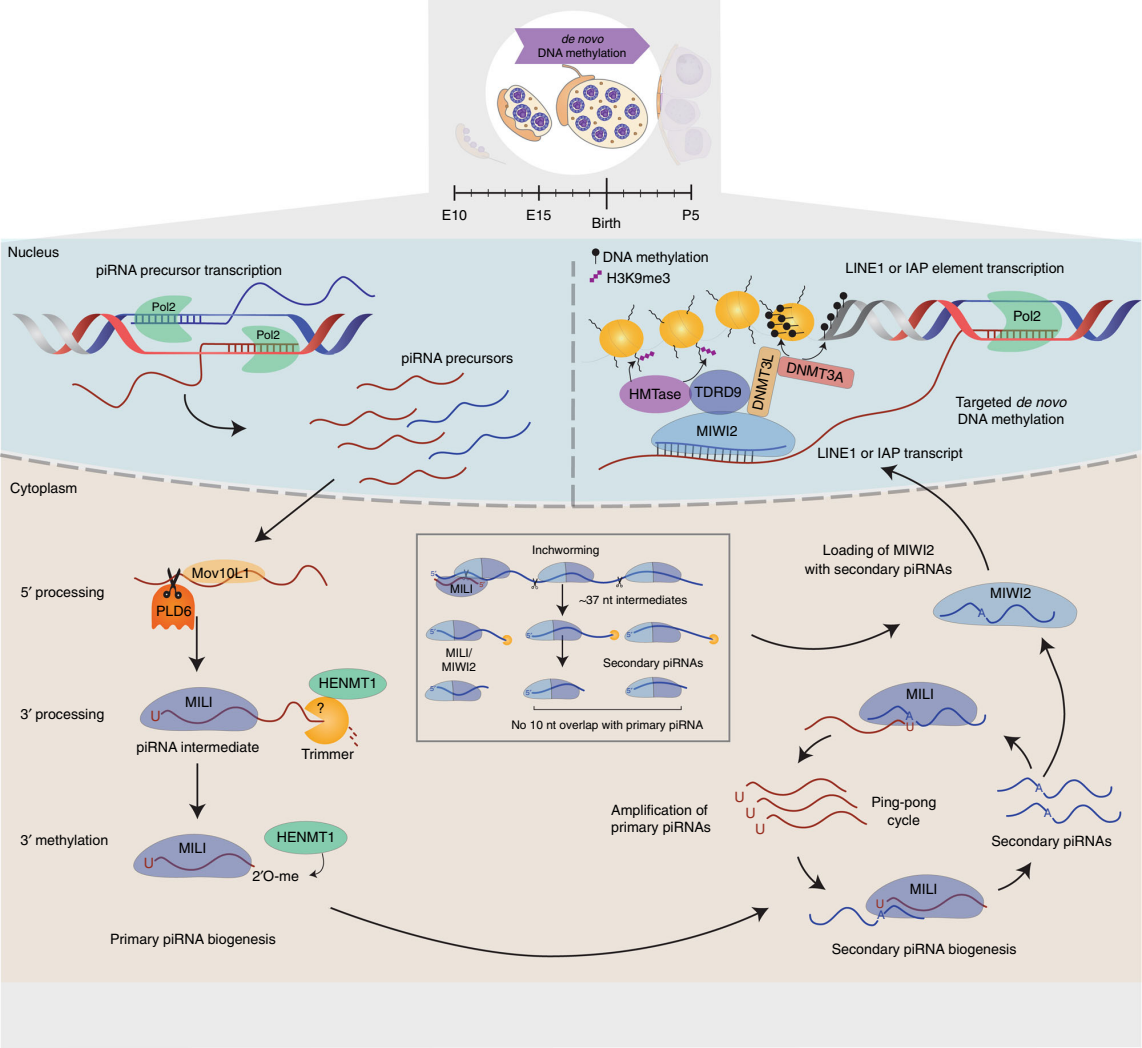
Biogenesis and function of pre-pachytene piRNAs in the mouse male germline. Pre-pachytene piRNAs are expressed during embryonic development from genomic loci that often display bidirectional transcription. piRNA precursors are exported to the cytoplasm and directed to the processing machinery. After 5′ end processing by PLD6, the piRNA intermediates associate with MILI. The 3′ ends of piRNA intermediates are then trimmed and 3′ methylated by Trimmer and HENMT1, respectively, to generate mature primary piRNAs bound to MILI. These complexes can then engage in the ping-pong cycle leading to the cleavage of complementary antisense transcripts and the production of secondary piRNAs bound to either MILI or MIWI2. Secondary piRNAs are characterized by a 10 nt overlap in their 5′ ends with primary piRNAs as a result of MILI-directed cleavage. Loading of MIWI2 with secondary piRNAs induces its translocation into the nucleus, where it functions in the transcriptional silencing of transposable elements (TEs). This involves the recruitment of the *de novo* DNA methylation machinery (DNMTs) as well as histone methyltransferases (HMTase) resulting in targeted DNA methylation and H3K9me3-mediated heterochromatinization of TE loci, such as LINEs and IAPs. An additional piRNA biogenesis mechanism, called inchworming, can produce MILI- and MIWI2-associated piRNAs from primary piRNA precursors without the characteristic 10 nt overlap with other piRNA species

Besides their association with different PIWI proteins, piRNAs are further distinguished based on their biogenesis. The biogenesis of primary piRNAs begins with the transcription of a single-stranded piRNA precursor molecule by RNA polymerase II (Pol II) from defined genomic locations termed piRNA clusters (Fig. 2). piRNA precursor molecules are then exported into the cytoplasm, which might be facilitated by Maelstrom (MAEL), a conserved HMG-box domain protein with RNA-binding activity^44^. Once in the cytoplasm, piRNA precursors associate with the RNA helicase MOV10L1, whose ATP-dependent unwinding activity is essential for piRNA biogenesis as illustrated by the increase in piRNA precursor transcripts and almost complete lack of mature piRNAs in MOV10L1 knockouts, or catalytically defective mutants^45, 46^. MOV10L1 is believed to fuel piRNA precursors into the biogenesis pathway by facilitating the interaction with PLD6 (MITOPLD), the endonuclease that generates the 5′ end of primary piRNAs^47, 48^. Intermediate fragments with a 5′ uridine (1U) are preferentially bound by PIWI proteins and thereby stabilized, leading to the strong 1U bias that is characteristic for primary piRNAs^49^. PIWI-bound piRNA intermediates then undergo 3′ end processing, which most likely involves exonucleolytic shortening by a 3′–5′ trimmer that has not been identified in mammals yet. The process is facilitated by Tdrkh, a Tudor and KH domain-containing protein, whose absence causes the accumulation of 31–37 nucleotide (nt) long intermediates^50^. The final length of piRNAs during 3′ end maturation is determined by the footprint of their associated PIWI protein, which protects the 3′ end from further trimming^51^. This generates size-specific piRNA populations for the different PIWI proteins with a mean length of 26 nt for MILI-, 28 nt for MIWI2-, and 30 nt for MIWI-bound piRNAs^32, 52, 53^. Mature 3′ ends are then 2′-O methylated by the RNA methyltransferase HENMT1 resulting in increased stability and binding to the PAZ domain of their PIWI protein^54^.

## Secondary piRNA biogenesis

Primary piRNAs can then guide the PIWI-mediated cleavage of antisense transcripts between nucleotide 10 and 11 to produce secondary piRNAs, a process known as the ping-pong cycle. Secondary piRNAs therefore have a defining 10 nt 5′ overlap with the primary piRNA that mediated the cleavage and often have adenine at their tenth position (10A)^53^. This 10A bias has long been thought to originate from complementarity to the 5′ uridine of primary piRNAs. However, base pairing at the first position is not necessary for efficient cleavage by Argonaute proteins^55^ and it appears that mammalian PIWI proteins rather have a preference for adenine at the t1 position that is not determined by base pairing^56^. Secondary piRNAs in turn can then fuel the production of the initiating primary piRNAs, thereby completing the ping-pong cycle. In the mouse, secondary piRNA production is driven by the slicer activity of MILI and most ping-pong cycles rely on homotypic MILI:MILI interactions with occasional heterotypic MILI:MIWI2 associations^57^. These heterotypic ping-pong interactions are however not reciprocal and the loading of MIWI2 with secondary piRNA is entirely dependent on MILI^53^. The disengagement of MIWI from the ping-pong cycle was shown to be due to active repression of the ping-pong cycle by RNF17, a Tudor family member, in meiotic cells. RNF17 knockout demonstrated that MILI:MIWI ping-pong interactions can occur *in vivo*, but result in spermatogenic arrest due to ping-pong-initiated piRNA responses against protein-coding genes^58^.

## Inchworm piRNA biogenesis

In addition to the primary and secondary piRNA biogenesis pathways, a separate mechanism termed “inchworming” fuels the production of MIWI2-associated piRNAs. Inchworming is initiated by MILI-loaded piRNAs that direct slicing of a target transcript. Instead of giving rise to only one conventional secondary piRNA, multiple evenly spaced, 37 nt long piRNA intermediates are formed in 5′–3′ direction from the initial cleavage product. These intermediates can associate with both MILI and MIWI2 and are trimmed at their 3′ ends to the typical length of 26 and 28 nt, respectively. Inchworm piRNAs do not have the characteristic 10 nt overlap of secondary piRNAs but display a strong 1U bias^59^.

MILI-associated piRNAs that initiate inchworming are in sense orientation to L1 elements and therefore lead to the production of large amounts of antisense inchworm piRNAs in contrast to the piRNAs produced in the ping-pong cycle. Thus, inchworming presents an amplification mechanism for antisense piRNAs that can silence L1 elements on the transcriptional or post-transcriptional level by associating with either MIWI2 or MILI, respectively^59^.

## Pre-pachytene piRNAs regulate DNA *de novo* methylation

MILI is the first PIWI protein to be expressed during development, starting at E13.5 in PGCs with a dynamic expression profile up until the round spermatid stage^30, 60, 61^. In contrast, MIWI2 is only expressed during a short period of time, starting at E14.5 until shortly after birth with protein levels being undetectable by P4^53, 61^. This expression window largely coincides with mitotic arrest of PGCs and *de novo* DNA methylation. Knockout of either MILI or MIWI2 leads to upregulation of TEs resulting in a spermatogenic arrest; however, not during the time of their joint expression, but only several days and cell divisions later in early pachytene spermatocytes^62^. piRNAs expressed during this period are termed pre-pachytene piRNAs and are characterized by a high occurrence of TEs in sense orientation, suggesting that most primary pre-pachytene piRNAs derive directly from TE transcripts^52, 53^. These piRNAs associate with MILI and drive the production of secondary piRNAs through recognition of antisense transcripts, which then associate with either MILI or MIWI2. This amplification method results in fine-tuning of the piRNA population and specific enrichment of those piRNAs that target the most active TEs.

While MILI is found exclusively in the cytoplasm in perinuclear granules called pi-bodies, MIWI2 is predominantly found in the nucleus but also localizes to cytoplasmic granules in the vicinity of pi-bodies, termed piP-bodies^63^. Translocation of MIWI2 into the nucleus is however dependent on its association with piRNAs and therefore on the slicer activity of MILI that is necessary to load MIWI2 with secondary piRNAs^53^. In contrast, the slicer activity of MIWI2 itself is dispensable for spermatogenesis, as catalytically inactive MIWI2 mutants show normal fertility and only modest upregulation of TEs^57^. This strongly suggests that MIWI2’s essential function during spermatogenesis defers from post-transcriptional gene silencing (PTGS) of TE transcripts in the cytoplasm and is rather related to its nuclear function. Indeed, MIWI2 is required for the establishment of *de novo* DNA methylation over TEs acting upstream of DNMT3L in PGCs^53^ (Fig. 2).

*De novo* methylation happens in two waves and many TEs manage to escape the first “generic” wave that occurs by E16.5. Interestingly, many of these evading TEs (mainly long interspersed element 1 (LINE1s) and intracisternal particle A (IAPs)) correspond to evolutionary young elements that also become activated as a result of MILI knockout and consequently unloaded MIWI2^64^. MIWI2 is believed to use antisense piRNAs as guides to detect nascent TE transcripts in the nucleus and recruits not only the *de novo* DNA methylation machinery^65^, but also histone methyltransferase to establish a repressive chromatin landscape especially across LINE1 elements^66^. Interestingly, preferential targeting of LINE1 elements by MIWI2 was also observed for DNA methylation. While loss of MIWI2 results in demethylation and derepression of mostly LINE1 elements, MILI deficiency causes DNA methylation defects across a wider range of TE families, including both LINE1 and LTR elements. This suggests that MILI can induce DNA methylation in a MIWI2-independent manner, however the underlying mechanisms are unknown^67, 68^. In contrast to these observations, a recent report showed greater disruption of DNA remethylation upon loss of Miwi2 compared to Mili, suggesting a MILI-independent piRNA biogenesis pathway in fetal male gonads^69^.

As mentioned above, spermatogenic arrest in MILI, MIWI2, or DNMT3L mutants does not occur until the pachytene stage but is characterized by a global derepression of LINE1 and IAP elements. It thus appears that DNA methylation is dispensable for TE silencing until the onset of meiosis due to additional transcriptional silencing mediated by H3K9me2^60^. This repressive histone mark, however, displays a dynamic pattern during spermatogenesis and completely disappears at the pachytene stage (Fig. 1b)^19^. Loss of H3K9me2 can be correlated with the expression of the histone methyltransferase G9a, which disappears at the leptotene stage preceding the H3K9me2 loss^70^. In DNA methylation mutants, the loss of repressive histone marks is concomitant with the gain of active marks such as H3K4me3. This results in the aberrant transcriptional activation of several classes of young repeat elements leading to the spermatogenic arrest at the pachytene stage^19^. Changes in the chromatin landscape of TEs perturb the meiotic process by relocalizing SPO11-mediated DSBs to repetitive elements. It is currently unclear whether the induced apoptosis is due to potential HR between non-allelic positions when repetitive elements participate in synapsis, or whether overall synapsis and repair is impaired due to a sequestration of the meiotic recombination machinery away from canonical recombination hotspots. Regardless of the underlying cause, this shows that even though spermatogenic arrest occurs several days and cell replications after the loss of DNA methylation, meiosis is sensitive to the transcriptional activation of TEs as these can disturb the dynamics of meiotic recombination. This appears to be a safeguard mechanism to eliminate cells in which *de novo* methylation of TEs has not been sufficient during reprogramming of PGCs. Beyond transposon control, pre-pachytene piRNAs have been implicated in the establishment of paternal imprinting by directing *de novo* DNA methylation to the differentially methylated region of the Rasgrf1 locus^71^.

Once MIWI2 expression ceases, MILI is the only PIWI protein expressed in male germ cells with its own expression levels decreasing gradually to very low or undetectable levels in pre-leptotene and leptotene spermatocytes until it increases again during the zygotene to pachytene transition^60^. Pre-pachytene piRNAs that engage with MILI during this time are involved in PTGS of TE transcripts that escape transcriptional gene silencing. This is supported by observations from conditional MILI knockout mice, in which the protein or its catalytic activity was removed at the differentiating spermatogonia stage. Despite intact DNA methylation in these animals, loss of MILI nevertheless results in spermatogenic arrest and high apoptosis^60^. Based on the lack of PIWI protein expression, it is suggested that pre-leptotene to late zygotene spermatocytes do not have an active piRNA pathway. This might be necessary to deplete the pool of pre-pachytene piRNAs from these cells to free up MILI proteins to engage with the new class of piRNAs about to be expressed.

## Pachytene piRNAs regulate TEs post transcriptionally

**Fig. 3:**
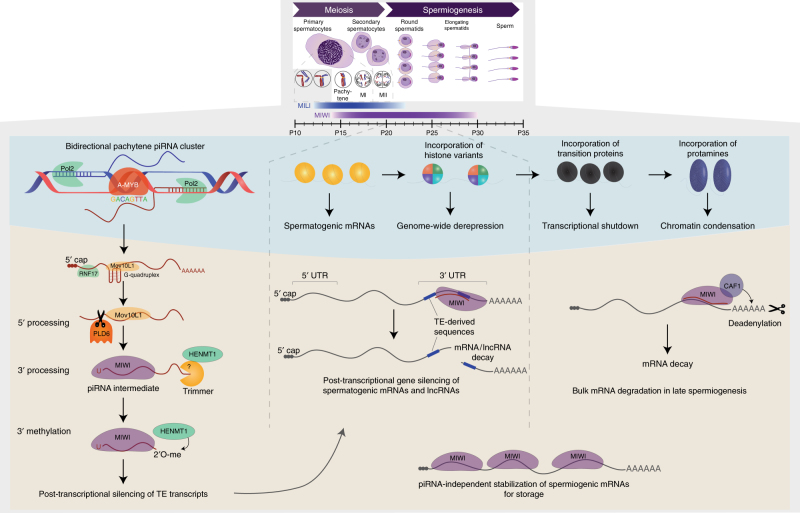
Multiple roles of action for pachytene piRNAs during mouse spermatogenesis. Pachytene piRNA expression starts during prophase of the first meiotic division when spermatocytes reach the pachytene stage. This expression is orchestrated by the transcription factor A-MYB, which drives expression of piRNA clusters and other piRNA pathway-related genes. Pachytene piRNAs are transcribed from distinct clusters in the genome often carrying bidirectional promoters. piRNA precursors are then processed in the same fashion as pre-pachytene piRNAs and mature pachytene piRNAs associate with either MILI or MIWI. Pachytene piRNAs engage in a myriad of functions throughout spermatogenesis, including the post-transcriptional silencing of TE transcripts but also non-TE-related functions. After meiosis, round spermatids undergo extensive epigenetic remodeling, resulting in a genome-wide derepression due to the incorporation of histone variants, followed by transition proteins that lead to a transcriptional shutdown, and finally replacement of histones with protamines leading to chromatin condensation. During this differentiation process, pachytene piRNAs regulate spermatogenic mRNAs and lncRNAs that become transcribed due to the genome-wide derepression. Furthermore, MIWI is involved in the piRNA-independent stabilization of spermiogenic mRNAs to allow storage and translation after the transcriptional shutdown. Toward later stages, pachytene piRNAs direct global mRNA degradation in association with MIWI, which recruits CAF1 to induce deadenylation resulting in mRNA decay

As the first wave of spermatocytes reaches the pachytene stage of meiosis, the transcription of the third PIWI family member MIWI starts together with a new class of piRNAs termed pachytene piRNAs^32–34^. Pachytene piRNAs are transcribed from large intergenic non-coding and 3′ untranslated regions of protein-coding gene loci^39, 72^, which have no unifying genomic sequence or motif definition and are fast evolving across mammals^73^. The majority of pachytene piRNAs (>80%) map uniquely to the genome, thereby allowing unambiguous identification of piRNA clusters^32^. Pachytene piRNA clusters can be either uni- or bidirectionally transcribed. Although there is a fewer number of bidirectional clusters annotated in the genome, they contribute to the majority of pachytene piRNAs (Fig. 3). Transcription of these clusters is facilitated by the ancestral transcription factor A-MYB. The resulting piRNA transcripts are 5′ capped and polyadenylated. However, A-MYB is not specific to piRNA clusters as it also drives its own expression as well as other members of the piRNA pathway in a positive feed-forward loop and thus cannot be the sole determining factor that distinguishes piRNA cluster transcripts from conventional mRNAs^74^. This was further supported by a recent study showing that the deletion of an A-MYB-binding site (GACAGTTA) from the promoter of a pachytene piRNA cluster resulted in a reduction but not complete depletion of piRNA precursor transcript levels and processed pachytene piRNAs^75^. This suggests that what directs piRNA precursors into the biogenesis pathway is more likely to be an RNA-based recognition signal and could involve the formation of G-quadruplex structures^45^. Similar to the observations in fetal gonocytes^63^, the piRNA pathway components involved in the processing and function of pachytene piRNAs also show distinct localization patterns to cytoplasmic nuage structures termed chromatoid bodies in mid-to-late meiotic spermatocytes and haploid spermatids^76, 77^.

In contrast to pre-pachytene piRNAs, pachytene piRNAs have a surprisingly low repeat content (<20%)^32^, that is in fact lower than the genome average^78^, thereby calling their importance in the regulation of TEs into question. However, it has been demonstrated that MIWI’s slicer activity is necessary for the accurate silencing of LINE1 elements in spermatocytes^79^. Nevertheless, the functional division between repeat-targeting and non-repeat-associated pachytene piRNAs still remains unclear.

Pachytene piRNA clusters have been shown to produce novel artificial piRNA sequences upon introduction of transgenic sequences^80^. A similar scenario is possible for new TE insertions, which has led to the suggestion that piRNA clusters may serve as “genomic sinks” to capture active TEs^44, 81^. However, in contrast to observations in Drosophila that show higher TE insertion rates into piRNA clusters^82^, no such bias has been described in mammals so far. It is therefore still unclear how the pachytene piRNA system adapts to newly invading repetitive elements and how quickly a new piRNA response can be installed.

## Non-repeat-related functions of pachytene piRNAs

MIWI remains actively transcribed until the round spermatid stage and is the only PIWI protein detected in late round and elongating spermatids^29^. These late stages of spermatogenesis are associated with major epigenetic changes and various functions have been implicated for non-repeat-related pachytene piRNAs. For example, the transitioning from histone- to protamine-bound DNA in round spermatids requires open and accessible chromatin, which leads to a genome-wide loosening of transcriptional control and the expression of spurious transcripts including many long non-coding RNAs (lncRNAs)^23, 83^. Pachytene piRNAs have been shown to be essential in targeting many of these mRNA and lncRNAs to regulate their expression via PTGS^84^ (Fig. 3). Interestingly, mutant mice for HENMT1, which lack methylation of piRNA 3′ ends leading to piRNA instability, exhibit a global loss of piRNAs resulting in the deregulation of meiotic transcripts^54^, further supporting the notion that piRNAs play a role in targeting and regulating meiotic gene expression on a post-transcriptional level^85^.

Recent work cloning a human piRNA cluster into the mouse and identifying mRNAs that are targeted by human ectopic piRNAs, shed light on the target specificity of this mechanism^86^. For a subset of ectopic piRNA sequences that overlapped with cleavage products profiled via 5′ RACE (Rapid amplification of cDNA ends), a perfect match between nucleotides 2–11 with a maximum of four mismatches between nucleotides 12–21 was detected. In support of this model, the observed male sterility phenotype of this mouse was explained by silencing of the germline-specific gene Dpy19L2 by one of the ectopically expressed human piRNAs. Double transgenic mice, expressing human piRNAs as well as the human Dpy19L2 gene that does not contain a target site for human piRNAs, were indeed fertile and able to produce offspring^86^.

During later stages of spermatogenesis in elongating spermatids, pachytene piRNAs were shown to direct the elimination of mRNAs. This was independent of the slicer activity of MIWI but required interaction with the deadenylase CAF1 to induce deadenylation and subsequent degradation of mRNAs^87^. Further functional implications for MIWI were made that are independent of associated piRNAs. In that case, MIWI binds to and stabilizes spermiogenic mRNAs that are stored for translation at later time points when transcription has been switched off^39, 44^.

Interestingly, one meiotic process, which has not been directly linked with piRNAs so far, is the meiotic silencing of unsynapsed chromosomes (MSUC). This mechanism is essential for meiosis, conserved across mammals, and results in transcriptional silencing of any chromosomes that are unpaired at the pachytene stage. One of the best studied examples for this is the meiotic silencing of the sex chromosomes in male germ cells due to their largely unpaired nature^88^. Studies have shown that conditional knockout of MILI after birth, therefore avoiding adverse phenotypes from MILI’s prenatal role, has no impact on the establishment of meiotic silencing across the X and Y chromosomes^89^. Interestingly, while piRNA clusters are absent from the X chromosome in the mouse, profiling of the piRNA repertoire in marmoset identified several piRNA cluster on the X chromosome, suggesting that these regions may escape meiotic silencing^81^.

## Additional mechanisms for transposon control

Periods of global epigenetic remodeling present TEs with an opportunity to become activated and retrotranspose, generating the need for the piRNA pathway in the male germline. Epigenetic reprogramming, however, is not unique to the male germline but also an essential process in female gametogenesis as well as preimplantation development in mouse embryos. Yet, the piRNA pathway is dispensable both in mouse oocytes as well as early embryos despite frequent upregulation of TEs^90–92^.

A possible explanation for the independence of the female germline in mice could be the lack of the fourth PIWI family member that exists in other mammals. PIWIL3 is found across eutherian mammals but is missing in the Muridae family comprising mice and rats. While its function remains largely elusive, it might hold some female germline-specific function as indicated by its exclusive expression in bovine oocytes^93^. In addition to PIWIL3, recent studies also found PIWIL1 expressed in both bovine oocytes and human fetal ovaries and suggest a role for piRNAs in the female germline of other mammals^94, 95^. A possible compensation for the loss of PIWIL3 in the Muridae family is the expression of an oocyte-specific Dicer isoform (DicerO) originating from a retrotransposon insertion. This insertion drives the expression of a shortened Dicer variant that is more efficient in processing double-stranded RNA into endogenous siRNAs^96^. In general, it has been shown that oogenesis is far more dependent on Dicer and other small RNA biogenesis factors^40, 97^. However, small RNAs, such as microRNAs, appear to function unperturbed throughout spermatogenesis. They are also not required for oogenesis and are in fact drastically downregulated during oocyte maturation, potentially to avoid competing effects between microRNA and endo-siRNA binding to AGO proteins^98, 99^.

Apart from germ cell development, early embryonic development is another developmental window during which the regulation of transposable elements is vital for proper cell differentiation^91, 92^. While some studies have implicated low levels of piRNA expression in embryonic stem cells (ESCs)^100^, their presence does not appear to be crucial. However, it has been shown that ESCs and early embryos show high levels of repeat activation and while the activation of repetitive elements is often part of the developmental program and helps to orchestrate gene expression, a more sophisticated fine-tuned system is required to ensure proper regulation^101–104^. TE silencing in early embryos mostly relies on transcriptional gene silencing mediated by H3K9me3 instead of DNA methylation^105–107^. One mechanism to specifically target transcriptional gene silencing to TEs is based on the large family of Cys2-His2 zinc finger (C2H2-ZF) proteins that form the largest family of transcription factors in both the mouse and human genome^108^. DNA binding is mediated via the use of up to several dozen C2H2-ZF domains in a combinatorial manner and results in large binding motifs. These proteins are often coupled to effector domains, the most common being the Krüppel-associated box (KRAB), and upon binding are called KRAB-ZFPs in mice or KZNFs in humans^109^. KRAB-ZFPs mediate transcriptional silencing by interacting with the corepressor protein KAP1 (KRAB-associated protein 1, also known as TRIM28), which serves as a scaffold domain to recruit chromatin modifiers like the histone lysine-methyl transferase SETDB1^110^.

KRAB-ZFPs are rapidly evolving and can be correlated to the emergence of species-specific TEs^111^. Indeed, an elegant study using transchromosomic mouse ESCs that carry one human chromosome was able to identify two human KZNFs that are necessary to repress two retrotransposon families that emerged relatively recently during human genome evolution^112^. Over time, repressed TEs will accumulate a sufficient number of mutations to render them inactive, making the KRAB-ZFP involvement in their recognition obsolete, which is reflected by the large number of KRAB-ZFP pseudogenes in the genome^111^. To date, only a few KRAB-ZFPs have been linked to a specific repeat element and their highly species-specific activities prohibit to draw conclusions from other model organisms. Assigning specific functions to KRAB-ZFPs in different species will remain challenging but the recent advance in identifying binding motifs and genomic target sites for numerous mouse and human KRAB-ZFPs will help to shed light onto this matter^113–116^. The KRAB-KAP1 pathway is an elegant example of co-adaptation between host genomes and their specific repeat content, which is dependent on gene duplication and mutation and therefore relatively slow. This could give highly active TEs the opportunity to cause major genomic damage until adaptation occurs. Our current understanding on how this adaptation is driven or directed is still very limited.

## Future challenges

Many parts and factors of the piRNA biogenesis pathway in mammals still remain unknown due to the lack of easy-to-use *in vitro* models to study this pathway. Recently published protocols for the differentiation of ESCs into PGCs or further into sperm^117, 118^ might allow the study of mammalian piRNAs in more detail. As of to date, most studies rely on the use of mouse knockout models and candidate genes are often chosen based on their described function in other organisms, such as Drosophila. However, piRNA pathway components identified in Drosophila are not always conserved in mammals, such as the rhino-deadlock-cutoff complex^119^, therefore making the mechanistic comparisons from insect to mammalian systems challenging. Without knowing the exact piRNA biogenesis pathway, other aspects such as identifying the signal that directs piRNA precursors to be processed, similar to piRNA trigger sequences in Drosophila^75^, remain a challenge.

## Conclusions

While silencing of TEs in the germline is the unifying function of piRNAs observed across all animals, piRNAs have evolved and adapted additional functions in mammalian spermatogenesis. This includes the establishment of proper DNA methylation including paternal imprints as well as regulating meiotic crossovers by defining the chromatin state. Furthermore, piRNAs have recently been shown to be essential for proper spermatogenesis by controlling and orchestrating the transcriptional program via the regulation of spermatogenic mRNAs on both transcriptional and post-transcriptional levels.
